# Results from the National *Legionella* Outbreak Detection Program, the Netherlands, 2002–2012

**DOI:** 10.3201/eid2107.141130

**Published:** 2015-07

**Authors:** Jeroen W. Den Boer, Sjoerd M. Euser, Petra Brandsema, Linda Reijnen, Jacob P. Bruin

**Affiliations:** Regional Public Health Laboratory Kennemerland, Haarlem, the Netherlands (J.W. Den Boer, S.M. Euser, L. Reijnen, J.P. Bruin);; National Institute for Public Health and the Environment, Bilthoven, the Netherlands (P. Brandsema)

**Keywords:** Legionnaires’ disease, surveillance, epidemiology, prevention and control, *Legionella* spp., genotype, the Netherlands, pneumonia, outbreak detection, bacteria

## Abstract

In 2002, the National *Legionella* Outbreak Detection Program was implemented in the Netherlands to detect and eliminate potential sources of organisms that cause Legionnaires’ disease (LD). During 2002–2012, a total of 1,991 patients with LD were reported, and 1,484 source investigations were performed. Of those sources investigated, 24.7% were positive for *Legionella* spp. For 266 patients with LD, 105 cluster locations were identified. A genotype match was made between a strain detected in 41 patients and a strain from a source location. Despite the systematic approach used by the program, most sources of LD infections during 2002–2012 remained undiscovered. Explorative studies are needed to identify yet undiscovered reservoirs and transmission routes for *Legionella* bacteria, and improved laboratory techniques are needed to detect *Legionella* spp. in clinical samples with a high background of microbial flora (such as soil).

## Abstract 2

This program has provided insights into the transmission, diagnosis, source investigation, and genotypic strain characteristics of the disease.

Legionnaires’ disease (LD) is an acute pneumonia characterized by clinical symptoms and signs (e.g., cough, fever, lung infiltration observed on a chest radiograph) similar to those of pneumonias resulting from other pathogens. LD is caused by infection with *Legionella* spp. bacteria, which are most often transmitted to persons through inhalation of bacteria disseminated into the air as an aerosol from natural or man-made sources of water (*1*). The incubation period is 2–14 days. LD is thought to account for 2%–20% of all community-acquired pneumonias (*2*) and is fatal in ≈6%–11% of cases (*3**,**4*).

After a large outbreak of LD at a flower show in Bovenkarspel, the Netherlands, in 1999 (*5*), prevention and control of *Legionella* spp. infections became a national concern in the Netherlands, and legislation to prevent *Legionella* spp. in drinking water systems was introduced (*6**,**7*). This legislation obligated owners of aerosol-producing devices (e.g., shower heads and whirlpools), if third parties may be exposed to them, to conduct a risk analysis, develop a control plan, keep logs of control measures, and perform regular sampling for *Legionella* spp. contamination. In addition, in 2002, a National *Legionella* Outbreak Detection Program (NLODP) was implemented (*8*) on the basis of a report that LD outbreaks are often preceded and followed by small clusters of solitary cases (*9*). The aims of NLODP are early detection of small clusters of cases, identification of sources of infection, and implementation of early control measures to prevent additional LD cases or an outbreak. For evaluation of transmission pathways, infection sources are sampled, and genotypes of *Legionella* strains found in these samples are compared with those of clinical isolate(s) from the patient(s) associated with that source. To evaluate the findings of the NLODP during 2002–2012, we analyzed data to determine whether extensive investigation efforts could detect *Legionella* spp. in collected samples and conclusively identify environmental sources.

## Methods

### Patients

LD has been notifiable in the Netherlands since 1987. A case of LD is defined as laboratory-confirmed infection in a person having symptoms compatible with pneumonia or radiologic signs of infiltration. Laboratory evidence may be >1 of the following: isolation of *Legionella* spp. from respiratory secretions or lung tissue, detection of *L. pneumophila* antigen in urine, seroconversion or a >4-fold rise in antibody titers to *L. pneumophila* in paired acute- and convalescent-phase serum samples, a high antibody titer to *L. pneumophila* in a single serum sample, and direct fluorescent antibody staining of the organism or detection of *Legionella* DNA by PCR in respiratory secretions or lung tissue. In the Netherlands, microbiologic laboratories involved in the diagnosis and treatment of patients with pneumonia are requested to send available clinical isolates of *Legionella* spp. to the *Legionella* Source Identification Unit (LSIU), a part of the NLODP. LD cases in persons who had been outside the country for >5 of 9 days before disease onset were defined as nondomestic cases and excluded from the analyses. Cases in persons who stayed in a hospital or other health care setting (e.g., nursing home or rehabilitation center) for >1 day during the 2–14 days before symptom onset were defined as nosocomial cases.

### Source Identification and Cluster Detection

Potential sources of infection were identified by Municipal Health Services (MHS) public health physicians and nurses, who used a standardized questionnaire to interview patients or relatives (Technical Appendix 1). The interview focused on tracking each patient’s exposure to potential sources of infection during the 2–14 days before symptom onset. All potential sources of infection were recorded in a database by the LSIU and used to identify clusters of LD cases by location and date. Each new LD case in this database was examined to determine if reported potential sources were linked to other LD cases. Because outbreaks of Legionnaires’ disease are often preceded and followed by small clusters of solitary cases (*9*), an arbitrary cluster definition was constructed that defined 2 types of clusters: location and geographic. A location cluster, which may represent a local contamination, was defined as cases reported within 2 years of each other in >2 persons who were reported to have been exposed to the same potential source of infection during the 2–14 days before symptom onset. A geographic cluster was defined as cases in >3 persons who lived <1 km apart and whose infections were reported within 6 months of each other. The concept of a geographic cluster was constructed to identify sources that patients were exposed to but unaware of (e.g., cooling towers). Patients could belong to >1 cluster. Data from the location cluster of the LD outbreak in Amsterdam in 2006 (*7*) were excluded from our analyses. 

### Sampling Procedure

As part of the NLODP, the LSIU is available to each MHS to collect samples from potential sources of *Legionella* infection for reported domestic LD cases. During 2002–2006, all identified potential sources of infection were investigated. However, because of budgetary reasons, after June 1, 2006, potential sources were investigated only if >1 of 4 sampling criteria was met: 1) a patient-derived isolate of *Legionella* spp. (from respiratory secretions or lung tissue) was available; 2) a location cluster was identified; 3) a geographic cluster was identified; or 4) the patient had stayed in a hospital or other health care setting during the incubation period. For geographic clusters, efforts were focused on identifying yet undiscovered potential sources (e.g., cooling towers near patients’ residences). If >1 of the 4 sampling criteria was met, trained LSIU laboratory staff collected water and swab samples from identified potential sources when possible. For each location, sampling points were selected by LSIU staff in cooperation with the facility’s technical team (when a team is available), and a comprehensive collection of water and swab samples was obtained from that location for further analysis.

### Laboratory Investigations

Samples collected during the source investigation were analyzed for the presence of *Legionella* spp. (for an extensive description, see Technical Appendix 2). All *L. pneumophila* serogroup 1 (SG1) strains (clinical and environmental) were subsequently genotyped by sequence-based typing, as recommended by the ESCMID Study Group for Legionella Infections (*10**–**12*), and further determined by using the Dresden panel of monoclonal antibodies (*13*). The sequence-based typing profiles of the patient isolates were compared with those of the environmental strains found in samples of potential sources.

### Statistical Analyses

Comparisons were made by using independent samples *t*-tests, nonparametric Mann-Whitney U-test, 2-tailed χ^2^ tests (proportions), and linear regression analyses (trends over time). All analyses were performed with PASW Statistics 8.0 (SPSS Inc., Chicago, IL, USA).

## Results

### Patients

During August 2002–August 2012, a total of 2,796 LD cases were reported in the Netherlands, 805 (28.8%) of which were nondomestic (Figure). These travel-associated cases were excluded from the analyses, resulting in 1,991 reported possible domestic LD cases (mean of 193 [SD 76] cases annually); 119 (6.0%) of these were characterized as nosocomial cases. Most patients (72%) for this period were male (Table 1). The median age of reported case-patients increased from 55.3 (range 26.4–78.3) years in 2002 to 62.5 (range 27.0–91.6) years in 2012 (Table 1; linear regression, p trend <0.001).

**Figure:**
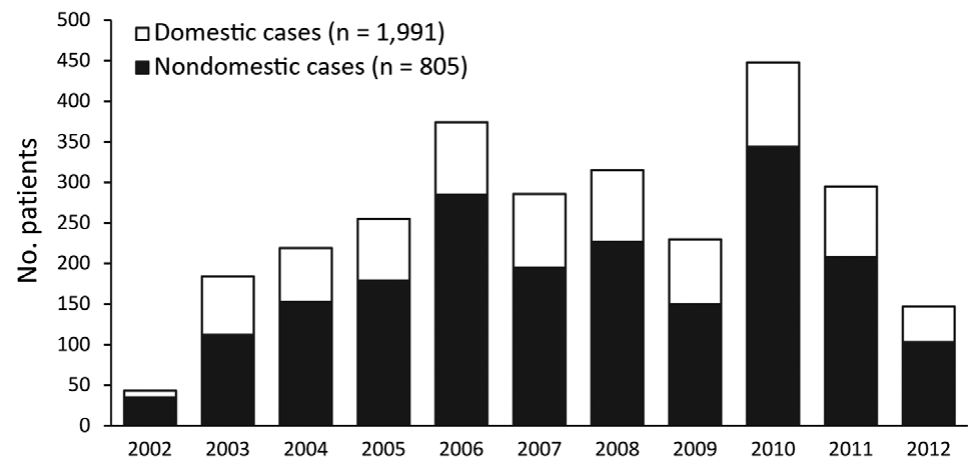
Legionnaires’ disease cases reported in the Netherlands, August 1, 2002–August 1, 2012. A total of 2,796 cases were reported; LD cases in persons who had been outside the country for >5 of 9 days before disease onset were defined as nondomestic cases and excluded from analyses. All other cases were classified as domestic.

**Table 1:** Number and demographic characteristics of patients with domestically acquired cases of Legionnaires’ disease, the Netherlands, 2002–2012*

Year	No. patients	Median age, y (range)	Male patients, no. (%)
2002	35	55.3 (26.4–78.3)	24 (68.6)
2003	112	57.4 (4.8–87.9)	73 (65.2)
2004	153	57.3 (21.3–88.1)	113 (73.9)
2005	179	59.1 (28.2–94.2)	132 (73.7)
2006	285	60.2 (17.0–90.1)	193 (67.7)
2007	195	59.1 (19.6–93.1)	153 (78.5)
2008	227	60.2 (11.0–98.1)	158 (69.6)
2009	150	61.3 (14.6–94.8)	106 (70.7)
2010	344	61.5 (23.2–94.1)	249 (72.4)
2011	208	62.2 (24.3–93.0)	155 (74.5)
2012	103	62.5 (27.0–91.6)	73 (70.9)
2002–2012	1,991	60.2 (4.8–98.1)	1,429 (71.8)

*Study period was August 1, 2002–August 1, 2012. Patients who had been out of the country for >5 of the 9 days before disease onset (n = 805) were excluded.

### Diagnostic Tests

The 1,991 LD cases were ascertained by 2,541 diagnostic tests (Table 2). Most cases were diagnosed by using urinary antigen tests (83.2%) or cultures (23.1%). Nosocomial LD cases (n = 119) were more often diagnosed by culture compared with community-acquired cases (37.0% vs. 22.2%; Pearson χ^2^ test, p<0.001). Nosocomial cases were more evenly distributed among male and female patients than were community-acquired cases (52.9% vs. 73.1% of cases in male patients, respectively; Pearson χ^2^ test, p<0.001).

**Table 2:** Characteristics of and test results for patients with domestically acquired Legionnaires’ disease, the Netherlands, 2002–2012*

Characteristic	Total, N = 1,991	Community acquired, n = 1,872	Nosocomial, n = 119	p value†
Patient demographics				
Age, y (SD)	60.2 (4.8–98.1)	60.0 (4.8–98.1)	68.9 (11.2–94.8)	<0.001
Male sex, no. (%)	1,429 (71.8)	1,366 (73.1)	63 (52.9)	<0.001
Diagnostic tests, no. (%)‡	2,541	2,380	162	NA
Culture‡	460 (23.1)	416 (22.2)	44 (37.0)	<0.001
Urinary antigen‡	1,656 (83.2)	1,567 (83.7)	89 (74.8)	0.012
Seroconversion‡	109 (5.5)	102 (5.4)	7 (5.9)	0.840
Direct immunofluorescence‡	3 (0.2)	3 (0.2)	0	NA
PCR‡	156 (7.8)	145 (7.7)	12 (10.1)	0.346
Single high titer‡	157 (7.9)	147 (7.9)	10 (8.4)	0.829

*Study period was August 1, 2002–August 1, 2012. For 1,499 patients, >1 diagnostic test was available. NA, not possible to calculate. †p value reflects the difference between patients with community-acquired Legionnaires’ disease and those with nosocomial Legionnaires’ disease. ‡Percentages reflect the proportion of patients for whom a diagnostic test result was available.

### Source Investigation

A total of 3,035 potential sources were identified for the 1,991 reported LD cases (mean of 1.5 [SD 1.0] potential sources per patient). Technical Appendix 2 Table 1 shows the distribution of the different types of reported sources. Using the NLODP sampling criteria, the LSIU sampled 1,418 unique potential sources (47% of 3,035 reported sources). Some sources were sampled >1 time, resulting in 1,484 source investigations performed during the study period. In 367 (24.7%) of these investigations, *Legionella* spp. were identified in >1 sample, but large variations were seen among the different source types (Table 3). In 30 investigations, >1 *Legionella* spp. was found, identified as *L. pneumophila* SG1 or *L. pneumophila* non-SG1 if no *L. pneumophila* SG1 was found (Table 3). The proportions before and after introduction of the 4 criteria for sampling on June 1, 2006, were similar: 24.6% vs. 25.2%, respectively.

**Table 3:** Sampling results (N = 1,484) by potential sources of infection for patients with Legionnaires’ disease, the Netherlands, 2002–2012*

Source type (no. samples)	Samples positive for *Legionella* spp., no. (%)				Samples negative for *Legionella* spp., no. (%)
	Total	*L. pneumophila *non-SG1	*L. pneumophila *SG1	*L.* non-*pneumophila*	
Wellness center (37)†	27 (73.0)	4 (10.8)	15 (40.5)	8 (21.6)	10 (27.0)
Hospital/health care setting (90)	46 (51.1)	5 (5.6)	23 (25.6)	18 (20.0)	44 (48.9)
Cooling tower (43)	19 (44.2)	8 (18.6)	9 (20.9)	2 (4.7)	24 (55.8)
Sports facility (29)	10 (34.5)	2 (6.9)	5 (17.2)	3 (10.3)	19 (65.5)
Swimming pool (40)	13 (32.5)	2 (5.0)	6 (15.0)	5 (12.5)	27 (67.5)
Hotel (20)	8 (40.0)	3 (15.0)	3 (15.0)	2 (10.0)	12 (60.0)
Holiday park (23)	5 (21.7)	1(4.3)	2 (8.7)	2 (8.7)	18 (78.3)
Residence (762)	155 (20.3)	30 (3.9)	21 (2.8)	104 (13.6)	607 (79.7)
Workplace (92)	19 (20.7)	8 (8.7)	2 (2.2)	9 (9.8)	73 (79.3)
Car wash/gasoline station (44)	6 (13.6)	1 (2.3)	NA	5 (11.4)	38 (86.4)
Garden center (86)	8 (9.3)	1 (1.2)	1 (1.2)	6 (7.0)	78 (90.7)
Campsite (28)	2 (7.1)	1 (3.6)	NA	1 (3.6)	26 (92.9)
Decorative fountain (23)	1 (4.3)	NA	NA	1 (4.3)	22 (95.7)
Other (167)	48 (28.7)	10 (6.)	10 (6.0)	28 (16.8)	119 (71.3)
Total (1,484)	367 (24.7)	76 (5.1)	97 (6.5)	194 (13.1)	1,117 (75.2)

*Study period was August 1, 2002–August 1, 2012. SG1, serogroup 1; NA, not possible to calculate. †Recreational facility offering spas, saunas, fitness equipment, massages, etc.

*L. pneumophila* SG1 was found in 97 (6.5%) investigations, *L. pneumophila* non-SG1 in 76 (5.1%), and *Legionella* spp. other than *L. pneumophila* in 194 (13.1%) (Table 3). The proportion of investigations in which *L. pneumophila* SG1 was found showed large variations among source types (Table 3). For instance, *L. pneumophila* SG1 was often detected in wellness centers (i.e., facilities offering spas, saunas, fitness equipment, massages, etc.) (40.5%); hospitals and health care settings (25.6%); and cooling towers (20.9%). However, *L. pneumophila* SG1 was not detected in investigated campsites, car wash or gasoline stations, or decorative water fountains and was detected in only a small proportion of investigated garden centers (1.2%). Residences were the most frequently sampled sources (51.3% of investigations); *L. pneumophila* SG1 was found in 21 (2.8%) of the 762 investigated residences (Table 3). Exclusion of source investigation data for the 119 nosocomial cases did not markedly change these results (Technical Appendix 2 Table 2).

### Clusters

The cluster definition used by NLODP resulted in 105 identified clusters, of which 98 (93.3%) were location clusters and 7 (6.7%) were geographic clusters. These clusters involved 266 patients with LD (Table 4; Technical Appendix 2 Figure). An average of 2.9 (range 2–11) patients with LD were associated with each cluster (some patients were part of multiple clusters). In 50 clusters (47.6%), patients from >1 MHS were involved. Garden centers were the most frequently identified cluster site (27 [25.7%] clusters), followed by hospitals and health care settings (17 [16.2%] clusters), residences (10 [9.5%] clusters), wellness centers (9 [8.6%] clusters), and hotels (7 [6.7%] clusters) (Table 5). For the 98 location clusters, 142 source investigations were performed (23 cluster locations were investigated >1 time during the study period). *Legionella* spp. were found in 56 (39.4%) of investigations. *L. pneumophila* SG1 was found in 28 (19.7%) investigations, *L. pneumophila* non-SG1 in 6 (4.2%), and *Legionella* spp. other than *L. pneumophila* in 22 (15.5%).

**Table 4:** Characteristics of 105 clusters reported for patients with Legionnaires’ disease (n = 266), the Netherlands, 2002–2012*

Characteristic	Value
Location clusters (%)†	98 (93.3)
Geographic clusters (%)‡	7 (6.7)
Mean no. patients per cluster (range)	2.9 (2–11)
No. multiple municipal health services involved (%)	50 (47.6)
Mean no. municipal health services involved (range)	1.7 (1–5)

*Study period was August 1, 2002–August 1, 2012.  †A location cluster is defined as cases reported within 2 years of each other in >2 persons who were reported to have been exposed to the same potential source of infection during the 2–14 days before symptom onset.  ‡A geographic cluster is defined as cases in >3 persons who lived <1 km apart and whose infections were reported within 6 months of each other.

**Table 5:** Cluster locations reported for 266 Legionnaires’ disease patients, the Netherlands, 2002–2012*

Reported cluster location	No. (%) clusters	Cluster type, no. (%)	
		Location†	Geographic‡
Garden center	27 (25.7)	27 (27.6)	0
Hospital/health care setting	17 (16.2)	17 (17.3)	0
Residence	10 (9.5)	4 (4.1)	6 (85.7)
Wellness center§	9 (8.6)	9 (9.2)	0
Hotel	7 (6.7)	7 (7.1)	0
Cooling tower	5 (4.8)	5 (5.1)	0
Holiday park	5 (4.8)	5 (5.1)	0
Swimming pool	4 (3.8)	4 (4.1)	0
Industrial complex	3 (2.9)	2 (2.0)	1 (14.3)
Car wash installation	3 (2.9)	3 (3.0)	0
Sports facility	2 (1.9)	2 (2.0)	0
Cruise ship	2 (1.9)	2 (2.0)	0
Other	11 (10.5)	11 (11.2)	0
Total	105 (100.0)	98 (100.0)	7 (100.0)

*Study period was August 1, 2002–August 1, 2012.  †A location cluster is defined as cases reported within 2 years of each other in >2 persons who were reported to have been exposed to the same potential source of infection during the 2–14 days before symptom onset.  ‡A geographic cluster is defined as cases in >3 persons who lived <1 km apart and whose infections were reported within 6 months of each other. §Recreational facility offering spas, saunas, fitness equipment, massages, etc.

### Strain Characteristics

For the 1,991 reported patients with LD, 392 clinical isolates of *Legionella* spp. (85% of 460 reported patients diagnosed by culture) were sent to LSIU by the participating microbiologic laboratories in the Netherlands. All *L. pneumophila* SG1 clinical isolates and environmental strains were genotyped by using sequence-based typing (*10**–**12*), and monoclonal antibody determination was performed (*13*) (Technical Appendix 2 Tables 3, 4).

### Matches

For the 392 patients with LD for whom a clinical isolate was available, 704 unique potential sources of investigation were identified (mean 1.8 [SD 1.2] sources per patient). For these sources, 478 investigations were performed, and *Legionella* spp. were found in a sample from 120 (25.1%) investigations.

Environmental strains were compared with the clinical isolate(s) from the patients associated with the sampled potential sources. During August 2002–August 2012, a total of 38 genotype matches were found for 41 patients with LD (3 matches involved 2 clinical isolates, and 35 matches involved 1 clinical isolate). For each patient with an isolate that was part of a genotype match, a mean of 1.9 (SD 1.6) potential sources of infection was identified. This mean was significantly higher than the mean 1.5 (SD 1.0) sources identified for patients whose clinical isolate could not be matched with an environmental strain (independent samples *t*-test, p<0.01). Table 6 shows the different types of sources from which the matching environmental strains were isolated. Most matches (15 [39%]) were with strains from hospitals or other health care settings, followed by those from residences (7 [18%]). A genotype match was found for 38 (31.7%) of 120 available clinical isolates that could be compared with an environmental strain (Technical Appendix 2 Table 5). For the 266 patients who were part of a cluster, 24 had clinical isolates that could be genotypically compared with environmental strains, and a genotype match occurred for 19 (79.2%) of these 24 patients.

**Table 6:** Genotypic matches (n = 38) from available isolates (n = 41) by source type reported for patients with Legionnaires’ disease, the Netherlands, 2002–2012*

Source type	No. (%) matches	No. (%) available isolates
Hospital/health care setting	15 (39.5)	17 (41.5)
Residence	7 (18.4)	8 (19.5)
Industrial complex	3 (7.9)	3 (7.3)
Swimming pool	2 (5.3)	2 (4.9)
Wellness center†	3 (7.9)	3 (7.3)
Hotel	2 (5.3)	2 (4.9)
Travel trailer	1 (2.6)	1 (2.4)
Whirlpool	2 (5.3)	2 (4.9)
Sports facility‡	1 (2.6)	1 (2.4)
Potting soil	1 (2.6)	1 (2.4)
Car wash installation	1 (2.6)	1 (2.4)
Total	38 (100.0)	41 (100.0)

*Study period was August 1, 2002–August 1, 2012. Data from the LD outbreak in Amsterdam in 2006 (7) are excluded from these data. †Recreational facility offering spas, saunas, fitness equipment, massages, etc. ‡This genotypic match was made with a clinical isolate collected during 2000 and an environmental strain collected in 2005.

## Discussion

During 2002–2012, a total of 1,991 patients with LD were reported in the Netherlands, and 1,484 source investigations were performed; 367 (24.7%) of the sources investigated were positive for *Legionella* spp. A total of 105 clusters were identified among 266 patients with LD. For 41 patients, a genotype match was found between the patient isolate and an environmental strain.

More than half of all source investigations were performed in residences, but only 20% of these investigations were positive for *Legionella* spp.; residences ranked tenth on the list of source types. A total of 43 cooling towers were investigated, ranking them third on the list of source types; >40% of those investigations were positive for *Legionella* spp. This well-known source of LD outbreaks should be considered often during source identification and investigation efforts performed by the MHS and LSIU. 

For each patient, a mean of 1.5 potential sources of infection were reported, and about half of the reported sources were sampled. Although several attributes are being used by the MHS to improve source investigation (e.g., an elaborate questionnaire and a geographic information system implemented in 2009 [https://lpgis.geoxplore.nl/webify/?app=lpgis_ggd]), the number of sources being sampled could be increased. When the genotypic matches were analyzed, the mean number of sources identified and investigated for the patients involved was considerably higher (1.9 sources per patient), suggesting that identification and investigation of more potential sources of infection by the MHS may increase the proportion of patients with LD for whom a likely source of infection can be established.

Garden centers ranked third (after residences and workplaces) on the list of the most frequently reported potential sources of LD infection; 26% of identified clusters were associated with a garden center, indicating that this source type is often visited by patients with LD during the 2–14 days before symptom onset. However, only 8 of 86 investigated garden centers were found positive for *Legionella* spp. during source investigations. Several studies have shown the presence of *Legionella* spp. in potting soil samples (*14**–**16*), and the use of amebal coculture techniques has shown promising results in recovering *L. pneumophila* SG1 sequence type (ST) 46 (the third most frequently found ST in clinical isolates) from samples with a high likelihood of microbial flora (*17*). At this time, potting soil samples collected by NLODP are not regularly being investigated by the amebal coculture technique. These findings suggest that potting soil samples from garden centers identified as potential sources of infection for patients with LD should be examined closely.

Notwithstanding the extensive efforts by NLODP collaborators, the number of *L. pneumophila* SG1 strains that could be derived from investigated potential sources was relatively low (114 strains over 10 years). Despite systematic methods of source identification by using a standardized questionnaire covering >20 source types, a source could not be confirmed in most cases. Although the questionnaire is regularly evaluated and adjusted on the basis of new insights concerning reported sources of infection, it primarily covers sources identified from the literature, possibly explaining the low success rate; actual sources of infection may not be captured in the questionnaire. This hypothesis is supported by the differences in genotype variation between clinical isolates and environmental strains: one third of all culture-positive patients with LD were infected by *L. pneumophila* SG1 ST47, a rare finding in environmental samples.

The experiences of NLODP show the importance of organizing a multidisciplinary collaboration in which MHSs, treating physicians, and microbiologic laboratories are represented and aware of the importance of different aspects of surveillance and source investigation for patients with LD. Our findings show the necessity of increasing awareness among various groups: physicians for diagnosis of LD, MHSs for extensive source identification, and laboratories for performance of adequate diagnostics and collection of clinical and environmental isolates. During 2002–2012, the number of reported patients with LD and the number of identified clusters of patients did not change dramatically, which may suggest the limited effects of NLODP. However, one could argue that this relatively stable number of patients with LD could have resulted from the program. Despite the rational, systematic approach used by NLODP during this decade, most sources of LD infections went undiscovered, stressing the need for evaluating other, yet unknown, potential sources of infection. Also, a need exists for further investment in improving laboratory techniques for detection of *Legionella* spp. in clinical samples with a high background of microbial flora such as soil.

**Technical Appendix 1.** Standardized questionnaire used by all Regional Public Health officers to assess reports of *Legionella* spp. Infections in the Netherlands.

**Technical Appendix 2.** Laboratory investigations of patients with Legionnaires’ disease, the Netherlands, 2002–2012.
