# Supplementary material for: Results from the National Legionella Outbreak Detection Program, the Netherlands, 2002–2012
Source: Emerg Infect Dis. 2015 Jul;21(7):1167–73. doi: 10.3201/eid2107.141130 (PMC4480379; doi:10.3201/eid2107.141130)
Supplement: Supplementary file 1 — Technical Appendix 1. Standardized questionnaire used by all Regional Public Health officers to assess reports of Legionella spp. Infections in the Netherlands. [file 14-1130-Techapp-s1.pdf]

Article DOI: http://dx.doi.org/10.3201/eid2107141130

Results from the National *Legionella* Outbreak Detection Program, the Netherlands, 2002–2012

Technical Appendix 1

Standardized questionnaire

Personal information

- Initials patient
- Date of birth
- Sex
- Postal code

Questions for treating physician

- First day of disease
- Was the patient admitted to a hospital?

If so, from which date?

- Did the patients suffer from a pneumonia?
- Was the pneumonia confirmed by X-ray?
- Did the patients die from the pneumonia?

If so, date of death:

Diagnosis

Was there a culture performed?
Date of culture:
Result of culture:
If positive, what *Legionella* spp. and serogroup?

Was there a urinary antigen test performed?
Date:
Result:

Was there a PCR test performed?
Date:

Result:

Was there a DFA test performed?
Date:
Result:

Was there a serological test performed?
Result (negative/single high titre/seroconversion):

Date of first blood sample:
Result:
Date of second blood sample:
Result:

Questions for the patient

1. Do you smoke? (yes/no/never/no stopped)

2. Do or did you suffer from a respiratory illness such as COPD or pneumonia in the past? (yes/no)

3. Do you suffer from a condition that influences your immune system (such as HIV-infection, AIDS, immunodeficiency)? (yes/no)

4. Do you use medication that suppresses the immune response (such as Prednison, chemotherapy) (yes/no)

5. Do you suffer from one of the following conditions:

    - o Chronic kidney disease
    - o Diabetes
    - o Organ transplantation
    - o Splenectomy
    - o Any form of cancer

Remarks on your condition?

6a. In what kind of home do you live?

- o Did not stay in own home during incubation period
- o Family home
- o Apartment
- o Elderly home
- o Nursing home
- o Home for the disabled
- o Other

6b. Name and address of the nursing home:

7a. Have you been attended to a hospital or other health care facility in the two weeks before you got sick?

If so, where and in what period?

8. Have you spend the night outside your own home (e.g. in a hotel) in the 14 days before you got sick?

9. If so, where?

- hotel
- campsite
- apartment or recreational home
- ship
- private residence
- holiday residence
- other:

9b. If it concerns a holiday residence, is it available for rent by others and/or are there shared facilities such as a swimming pool at the accommodation?

9c. In what period did you stay here?

9d. Name and address of residence, room number, phone number, tour operator.

9e. Have you been in contact with aerosols, other than in the bathroom of your own room/apartment/caravan (e.g. sauna, whirlpool, outdoor shower)?

10. Other accommodations

11. What is your profession?

12. What is the nature of your work?

13. What was your most frequently visited working address for the last 2 weeks?

14. Have you used a shower at your work in the two weeks before you got sick?

If so, where?

15. Have you been in contact with aerosols during your work in the two weeks before you got sick?

If so, where?

16. Have you used a shower in a sport accommodation in the two weeks before you got sick?

If so, where?

17. Have you visited a sauna in the two weeks before you got sick?

If so, where?

18. Have you used a shower in a solar studio/fitness/aerobics center in the two weeks before you got sick?

If so, where?

19. Have you used a swimming pool and/or shower in a swimming pool in the two weeks before you got sick?

If so, where?

20. Have you spent time in a whirlpool in the two weeks before you got sick?

If so, was this a private or public whirlpool?
If public, where?

21. Have you been at an event such as an exhibition, fair, or attraction park the two weeks before you got sick?

If so, where?

22. Have you visited a garden center in the two weeks before you got sick?

If so, where?

23. Have you visited a location with a fountain and/or sprinkler installation in the two weeks before you got sick?

If so, where?

24. Have you washed your car in a car wash installation in the two weeks before you got sick?

If so, where?

Other locations?

25. What temperature has been assigned to the boiler or heating system in your home? Have there been problems with the system in the last 14 days?

26. Have there been adaptations to the central heating system in your home in the last two weeks?

28. Have you worked in the garden and/or had contact with natural or potting soil in the two weeks before you got sick?

29. Have you used a garden hose, a high pressure hose, or an evaporator for plants and flowers in the two weeks before you got sick?

30. Other risk full activities?

31. Do you know people in your direct surroundings who have recently been admitted to a hospital with a pneumonia?

If so, which hospital, and what is the residence of this patient?

32. Do you have any idea where you have been infected with this Legionella-pneumonia?

If so, where?

Date	Symptoms	Incubation time (days)	Exposed to possible source
		14	
		13	
		12	
		11	
		10	
		9	
		8	
		7	
		6	
		5	
		4	
		3	
		2	
		1	
	First day of disease	0	
