# Supplementary material for: Results from the National Legionella Outbreak Detection Program, the Netherlands, 2002–2012
Source: Emerg Infect Dis. 2015 Jul;21(7):1167–73. doi: 10.3201/eid2107.141130 (PMC4480379; doi:10.3201/eid2107.141130)
Supplement: Supplementary file 2 — Technical Appendix 2. Laboratory investigations of patients with Legionnaires’ disease, the Netherlands, 2002–2012. [file 14-1130-Techapp-s2.pdf]

Article DOI: http://dx.doi.org/10.3201/eid2107141130

Results from the National *Legionella* Outbreak Detection Program, the Netherlands, 2002–2012

Technical Appendix 2

Laboratory Investigations

The water samples (500 ml) were concentrated by using membrane filtration. Filtration was done by vacuum filtration (550 bar) with the aid of a vacuum controller (Innotech Europe BV; Moergestel, the Netherlands). The scraping technique, as described in ISO 11731: 1998 annex A, have been used for removal of the organisms from the membrane. Residues were resuspended in 1 ml sterile water. Of this suspension, 100 µl samples were cultured without dilution and after 10-fold dilution on 2 media at 35°C, with increased humidity. The 2 media used were buffered charcoal yeast extract supplemented with α-ketoglutarate (BCYE-α) and (i) the antimicrobial drugs polymyxin B, cefazolin, and pimaricin; and (ii) the antimicrobial drugs polymyxin B, anisomysin, and vanomycin (Oxoid, Basingstoke, United Kingdom). In cases of bacterial overgrowth, cultures were repeated after pretreatment by heating for 30 minutes at 50°C. Swab samples were dispersed by immersion in 1 ml sterile water and cultured, as described above. First examination was done on day 2 of the incubation period (7–10 days). The examination of the media was performed with the aid of a dissection microscope. Both patient and environmental *Legionella* isolates were serotyped by using commercially available kits containing antisera against *L. pneumophila* serogroups 1–14, *L. longbeachae* 1 and 2, *L. bozemanii* 1 and 2, *L. dumoffii, L. gormanii, L. jordanis, L. micdadei,* and *L. anisa* (Legionella latex test, Oxoid Limited, Hampshire, England; Legionella antisera “Seiken,” Denka Seiken Co. Ltd, Tokyo, Japan).

Technical Appendix Table 1. Potential sources of infections reported by patients with Legionnaires' disease (n = 1,991), the Netherlands, 2002–2012

Reported potential source of infection	No. (%)
Residence	1,746 (57.5)
Workplace	203 (6.7)
Garden center	158 (5.2)
Hospital/other health care setting	108 (3.6)
Car wash/gasoline station	106 (3.5)
Sports facility	90 (3.0)
Swimming pool	68 (2.2)
Decorative fountain	62 (2.0)
Cooling tower	57 (1.9)
Campsite	56 (1.8)
Hotel	54 (1.8)
Holiday park	51 (1.7)
Dental practice	30 (1.0)
Wellness center	29 (1.0)
Other	217 (7.1)
Total	3,035 (100.0)

Technical Appendix Table 2. Potential sources of infections reported by patients with community-acquired Legionnaires' disease (n = 1.872), the Netherlands, 2002–2012*

Reported potential source of infection	No. (%)
Residence	1,673 (59.6)
Workplace	198 (7.0)
Garden center	153 (5.4)
Car wash/gasoline station	105 (3.7)
Sports facility	87 (3.1)
Swimming pool	66 (2.3)
Decorative fountain	61 (2.2)
Cooling tower	52 (1.9)
Campsite	55 (2.0)
Hotel	51 (1.8)
Holiday park	49 (1.7)
Dental practice	28 (1.0)
Wellness center	27 (1.0)
Other	204 (7.3)
Total	2,809 (100.0)

*Nosocomial cases (n = 119) are not included in these analyses.

Technical Appendix Table 3. Distribution of clinical isolates and environmental strains of *Legionella pneumophila* SG 1 by sequence type, the Netherlands, 2002–2012*

Legionella pneumophila SG1	Total (n = 469)		Clinical isolates (n = 355)		Environmental strains (n = 114)	
ST	No. (%)	%	No.	%	No.	%
47	126	26.9	125	35.2	1	0.9
1	65	13.9	18	5.1	47	41.2
62	44	9.4	44	12.4	–	–
42	24	5.1	11	3.1	13	11.4
9	20	4.3	13	3.7	7	6.1
23	20	4.3	17	4.8	3	2.6
46	20	4.3	19	5.4	1	0.9
37	19	4.1	12	3.4	7	6.1
45	14	3.0	14	3.9	–	–
82	8	1.7	8	2.3	–	–
59	5	1.1	1	0.3	4	3.5
109	5	1.1	5	1.4	–	–
146	4	0.9	3	0.8	1	0.9
334	4	0.9	2	0.6	2	1.8
48	3	0.6	2	0.6	1	0.9
110	3	0.6	2	0.6	1	0.9
115	3	0.6	3	0.8	–	–
170	3	0.6	3	0.8	–	–
207	3	0.6	3	0.8	–	–
479	3	0.6	3	0.8	–	–
534	3	0.6	1	0.3	2	1.8

Legionella pneumophila SG1	Total (n = 469)		Clinical isolates (n = 355)		Environmental strains (n = 114)	
ST	No. (%)	%	No.	%	No.	%
7	2	0.4	–	–	2	1.8
20	2	0.4	2	0.6	–	–
75	2	0.4	2	0.6	–	–
117	2	0.4	2	0.6	–	–
177	2	0.4	2	0.6	–	–
188	2	0.4	1	0.3	1	0.9
345	2	0.4	1	0.3	1	0.9
444	2	0.4	1	0.3	1	0.9
493	2	0.4	1	0.3	1	0.9
953	2	0.4	1	0.3	1	0.9
Other STs	45	9.6	33	9.3	12	10.5
Total	469	100.0	355	100.0	114	100.0

*Only sequence types that were available >2 times in the total collection (n = 469) are included in this table. SG, serogroup; ST, sequence type.

Technical Appendix Table 4. Distribution of clinical isolates and environmental strains of *Legionella pneumophila* by phenotype, 2002–2012*

		Total		Clinical isolates (n = 387)		Environmental strains (n = 214)	
Serogroup	mAb subgroup	No.	%	No.	%	No.	%
1	Allentown/France	210	34.9	201	51.9	9	4.2
1	Benidorm	67	11.1	50	12.9	17	7.9
1	Knoxville	45	7.5	37	9.6	8	3.7
1	Philadelphia	41	6.8	32	8.3	9	4.2
	(MAb3/1 positive)	(363)	(60.4)	(320)	(82.7)	(43)	(20.1)
1	Bellingham	26	4.3	9	2.3	17	7.9
1	Camperdown	6	1.0	–	–	6	2.8
1	Heysham	1	0.2	1	0.3	–	–
1	OLDA	57	9.5	24	6.2	33	15.4
1	Oxford	17	2.8	1	0.3	15	7.0
	(mAb 3/1 negative)	(106)	(17.6)	(35)	(9.0)	(71)	(33.2)
1	(all subgroups)	(469)	(78.0)	(355)	(91.7)	(114)	(53.3)
2		15	2.5	8	2.1	7	3.3
3		28	4.7	9	2.3	19	8.9
4		1	0.2	–	–	1	0.5
5		16	2.7	1	0.3	15	7.0
6		9	1.5	3	0.8	6	2.8
2–14		2	0.3	1	0.3	1	0.5
7–14		61	10.1	10	2.6	51	23.8
Total		601	100.0	387	100.0	214	100.0

*mAb, monoclonal antibody; mAb subgroups Allentown/France, Benidorm, Knoxville, and Philadelphia all belong to the mAb 3/1 positive group. mAb subgroups Bellingham, Camperdown, Heysham, OLDA, and Oxford all belong to the 3/1 negative group. The 5 clinical isolates determined to be *Legionella* non–*pneumophila* (2 *L. longbeachae* isolates, 1 *L. micdadei*, 1 *L. dumoffi*, and 1 *L. anisa*) are not presented here.

Technical Appendix Table 5. *Legionella pneumophila g*enotypes for the 38 genotypic matches for patients with Legionnaires' disease, the Netherlands, 2002–2012*

Genotype	No. (%)
Legionella pneumophila SG1	*26 (68)*
ST1	7 (18)
ST42	3 (8)
ST23	2 (5)
ST37	2 (5)
ST177	2 (5)
ST334	2 (5)
ST9	1 (3)
ST47	1 (3)
ST48	1 (3)
ST110	1 (3)
ST345	1 (3)
ST444	1 (3)
ST534	1 (3)
ST953	1 (3)
L. pneumophila SG3	3 (8)
L. pneumophila SG6	1 (3)
L. pneumophila SG7-14	7 (18)

Genotype	No. (%)
L. longbeachae	1 (3)

*SG, serogroup; ST, sequence type.

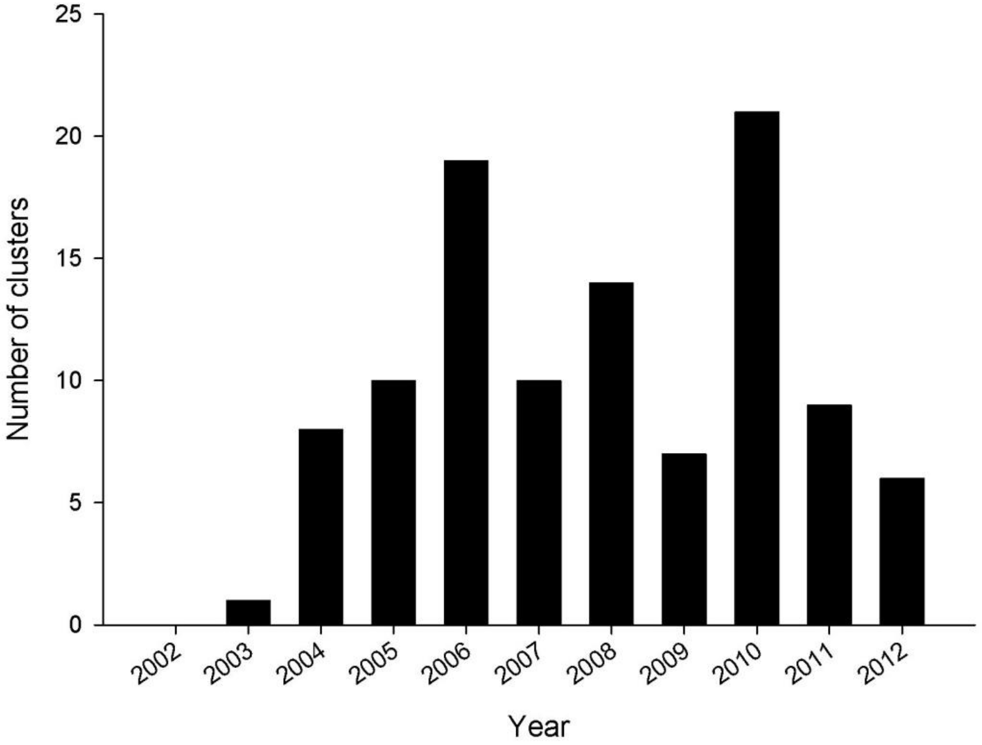

Technical Appendix Figure. Number of clusters of Legionnaires’ disease patients in the Netherlands by year, 2002–2012.
